# Assembly and activation of the NLRP3 inflammasome and cytokine quantification in response to exercise in adults with different metabolic conditions: a systematic review

**DOI:** 10.3389/fspor.2025.1602208

**Published:** 2025-07-21

**Authors:** Salma Verenice Cristerna-Huerta, Melissa Vega-Burgueño, Marcela de Jesús Vergara-Jiménez, Erika Martínez-López, Elisa Barrón-Cabrera

**Affiliations:** ^1^ Facultad de Ciencias de la Nutrición y Gastronomía, Universidad Autónoma de Sinaloa, Culiacán, México; ^2^ Instituto de Nutrigenética y Nutrigenómica Traslacional, Departamento de Biología Molecular y Genómica, Centro Universitario en Ciencias de la Salud, Universidad de Guadalajara, Guadalajara, México

**Keywords:** ASC, caspase-1, exercise training, NLRP3 inflammasome, NLRP3 physical activity

## Abstract

**Background:**

The NLRP3 inflammasome is a molecular structure involved in inflammation and innate immune response, its overexpression has been associated with the development of several diseases. Physical exercise plays an important role in regulating systemic inflammation, however different types of exercise seem to have a different effect in the regulation of the NLRP3 inflammasome.

**Objective:**

To provide updated information related to the effect of different types and training durations of exercise on NLRP3 inflammasome complex, IL-1β—IL-18 cytokines quantification in adults with different metabolic conditions.

**Methods:**

The Preferred Reporting Items for Systematic Reviews and Meta-Analyses (PRISMA) methodology for manuscript research and preparation was followed using PubMed, Science Direct, SpringerLink, Scopus and Google Scholar databases for literature review. Out of 1,514 articles identified, only 11 articles fulfilled the inclusion criteria.

**Results:**

Moderate-intensity aerobic exercise and moderate resistance exercise seems to significantly decreased concentrations of the cytokine IL-1β, NLRP3 protein and caspase-1, as well as *ASC* and *NLRP3* gene expression. High-intensity aerobic exercise exerted the opposite effects by increasing *NLRP3* gene expression and the cytokines IL-1β and IL-18.

**Conclusion:**

Resistance, aerobic and combined exercise (≥8 weeks) were linked to downregulated key NLRP3 inflammasome components across diverse populations. These results support exercise as a safe and effective strategy to modulate NLRP3-driven inflammation. Notably, evidence suggest that resistance and combined modalities showed superior efficacy in reducing both gene and cytokine levels. This positions structured exercise as a valuable tool in managing chronic low-grade inflammation.

## Introduction

NLRP3 inflammasome formation is initiated by the nucleotide oligomerization domain (NOD)-like receptors (NLR), which are key in the regulation of the innate immune system and the recognition of diverse intracellular pathogens such as the damage-associated molecular patterns (DAMPs), pathogen-associated molecular patterns (PAMPs), danger signals, caspase-1 activity, and the secretion of IL-18 and IL-1β cytokines (1).

The NLRs are classified according to the protein binding domains located in their amino-terminal region: (1) NOD composed of 6 members, (2) IPAF or NLRC consisting of 2 members, and (3) Nucleotide-binding oligomerization domain, Leucine rich Repeat and Pyrin domain Containing (NLRP), for which currently 14 members are recognized. The NRLPs are associated with the formation of inflammasomes, which are molecular complexes that serve as sensors and mediators of systemic inflammation (2).

There are diverse isoforms of NLRP, including NLRP1 to NLRP14. However, NLRP3, also known as cryopyrin, is the most studied and characterized cause their close relationship with chronic diseases (3). NLRP3 inflammasome has three components: (1) The NLRP3 protein which contains a central nucleotide-binding oligomerization, (2) an ASC adaptor protein and (3) the procaspase-1 (4).

The activation of the NLRP3 inflammasome requires two independent signals (5). The first signal is considered priming and promotes the transcription of NLRP3, pro-IL-1β, and pro-IL-18 proteins form. The second signal is activation and this results in the oligomerization and assembly of the NLRP3 inflammasome, promoting the cleavage of caspase-1, which, in turn, cleaves pro-IL-1β and pro-IL-18 cytokines into their mature forms.

The activation of NLRP3 inflammasome-dependent caspase-1 is also implicated in a form of inflammatory cell death known as pyroptosis (1). Pyroptosis is characterized by cellular inflammation, plasma membrane rupture due to water influx, and the release of proinflammatory cellular contents (6).

NLRP3 inflammasome serves as the central axis of innate immunity, regulating the secretion of proinflammatory cytokines and playing an indispensable role in modulating inflammatory responses (7). Inflammation stands as a key characteristic influencing the development and progression of numerous diseases (8). The NLRP3 inflammasome can act both as a sensor and contributor in the pathogenesis of obesity (9), certain types of cancer, metabolic diseases such as diabetes (10), atherosclerosis, cardiovascular diseases (11), and neurodegenerative diseases (12). Unlike other inflammasomes, NLRP3 inflammasome response to numerous stimuli, including environmental factors like diet and physical exercise (13).

On the other hand, the majority of individuals affected by chronic diseases and conditions related to uncontrolled inflammasome activation share similar lifestyle behaviors, such as being overweight or obese, unhealthy dietary habits, and physical inactivity (14). These behaviors are major contributors to diseases causing significant mortality worldwide. Physical inactivity alone contributes to 9% of global deaths (15), despite the well-known benefits of exercise in disease related to inflammatory pathways (16).

A study conducted by Abd El-Kader & Al-Ahreef demonstrated that exercise has an anti-inflammatory effect by reducing proinflammatory markers such as TNF-α, IL-6, and CRP, accompanied by an increase in IL-10 in blood samples from older adults (17). Furthermore, scientific evidence suggests that exercise may regulate inflammation through the NLRP3 inflammasome (18). However, the optimal intensity, frequency, and duration of training to achieve the best benefits in inflammation indicators associated with the NLRP3 inflammasome are still unknown.

Therefore, the aim of this systematic review is to provide updated information related to the effect of different types and training durations of exercise on the NLRP3 inflammasome complex, quantification of cytokines IL-1β—IL-18 in adults with different metabolic conditions to provide specific recommendations on which type of exercise contributes to a significant reduction in NLRP3 inflammasome levels.

## Methods

This systematic review was conducted according to the Preferred Reporting Items for Systematic Reviews and Meta-Analysis (PRISMA) guidelines, as per its latest update (19).

### Search strategy

PubMed, Science Direct, SpringerLink, Scopus and Google Scholar were employed for a systematic literature search that were published between January 1st, 2020 to January 31th, 2025. For the Google Scholar database, Publish or Perish software was used to optimize the search. The search was restricted to studies published in English. The following search filters were used: clinical trial, human species, adults aged 18 years or older. The search terms used, either individually or in combination, were selected based on PubMed MeSH terms: “physical exercise”, “exercise training”, “inflammasome”, “NLRP3 inflammasome”, “adults”, “metabolic diseases”, “NLRP3”, “ASC”, “caspase-1”, “pro-caspase-1”, “IL-1β”, and “IL-18”. A manual search of the reference lists of selected original articles was conducted to identify additional studies.

### Inclusion and exclusion criteria

Inclusion criteria: Randomized clinical trials, case-control studies, published in the last 5 years, written in English, include data on the physical exercise program (duration, intensity, frequency), quantitatively assess at least one of the NLRP3 inflammasome-associated components (NLRP3, ASC, CASPASE-1, IL-1β and IL-18).

Exclusion criteria: reviews, studies based on animal models or *in vitro* cellular models, studies without exercise intervention (this refers to unstructured exercise with weekly sessions, defined intensity, and a duration of at least 4 weeks), studies without a structured physical exercise program, single-session interventions, marathons, competitions, races, incomplete data on the variables of interest and studies with a population sample under 18 years of age.

Data are shown following the population, intervention, comparison, outcome, and study design (PICOS) approach:

• Population: Adults aged ≥18 years old, without restrictions on gender or health conditions.
• Intervention: Various types of exercise, including isometric grip, resistance exercise, aerobic exercise, combined aerobic and resistance exercise, with a a frequency of at least two sessions per week, with a minimum duration of 20 min per session, exercise intensity between 30% and 100% of maximum heart rate, minimum duration of 4 weeks of intervention.
• Comparison: Control group without exercise compared to an exercise group, under the same habitual conditions.
• Outcome: Indicators related to the gene expression and protein concentrations of the NLRP3 inflammasome, including concentrations of NLRP3, ASC, pro-caspase-1, caspase-1, IL-18, and IL-1β, measured in peripheral blood (plasma, serum, or peripheral blood mononuclear cells).
• Study Design: Randomized controlled trials (RCTs), cohort and case-control studies published in English language.

Titles and abstracts of identified articles were independently reviewed. Following preliminary screening, full-text articles were examined according to the selection criteria. For this systematic review, studies were grouped according to the type of exercise performed: aerobic exercise, resistance exercise, and combination of aerobic and resistance exercise, as well as training duration exercise (≤12 weeks or ≥13 weeks).

### Data extraction

The following characteristics of the selected studies were extracted: Author, publication year, country where the study was conducted, health status, age, population characteristics, biological sample, pharmacological and/or dietary treatment, and exercise intervention details (type, method, intensity, frequency, duration). Means and standard deviations of indicators associated with inflammasome activation (NLRP3, ASC, caspase-1, IL-1β, IL-18) were also extracted.

### Risk of bias assessment

The methodological quality of the selected studies was assessed using the “Cochrane Tool for Risk of Bias” (20). This tool evaluates seven aspects of potencial bias: (1) random sequence generation (selection bias); (2) allocation concealment (selection bias); (3) blinding of participants and personnel (performance bias); (4) blinding of outcome assessment (detection bias); (5) incomplete outcome data (attrition bias); (6) selective reporting (reporting bias); (7) other potencial sources biases.

Each aspect was rated as “low risk”, “high risk”, or “unclear risk” of bias, according to the criteria described in the Cochrane tool. For scoring purposes, only domains rated as “low risk” were assigned one point; aspects rated as “high” or “unclear” received zero points. Thus, the maximum possible score per study was 7. Based on the total score, each study could be classified as high quality (5–7 points), moderate quality (3–4 points), or low quality (0–2 points) (20).

To ensure consistency, the risk of bias was independently evaluated by two reviewers. Discrepancies between reviewers were resolved through discussion and consensus; if agreement could not be reached, a third reviewer was consulted to make the final decision.

## Results

### Study selection

**Figure 1:**
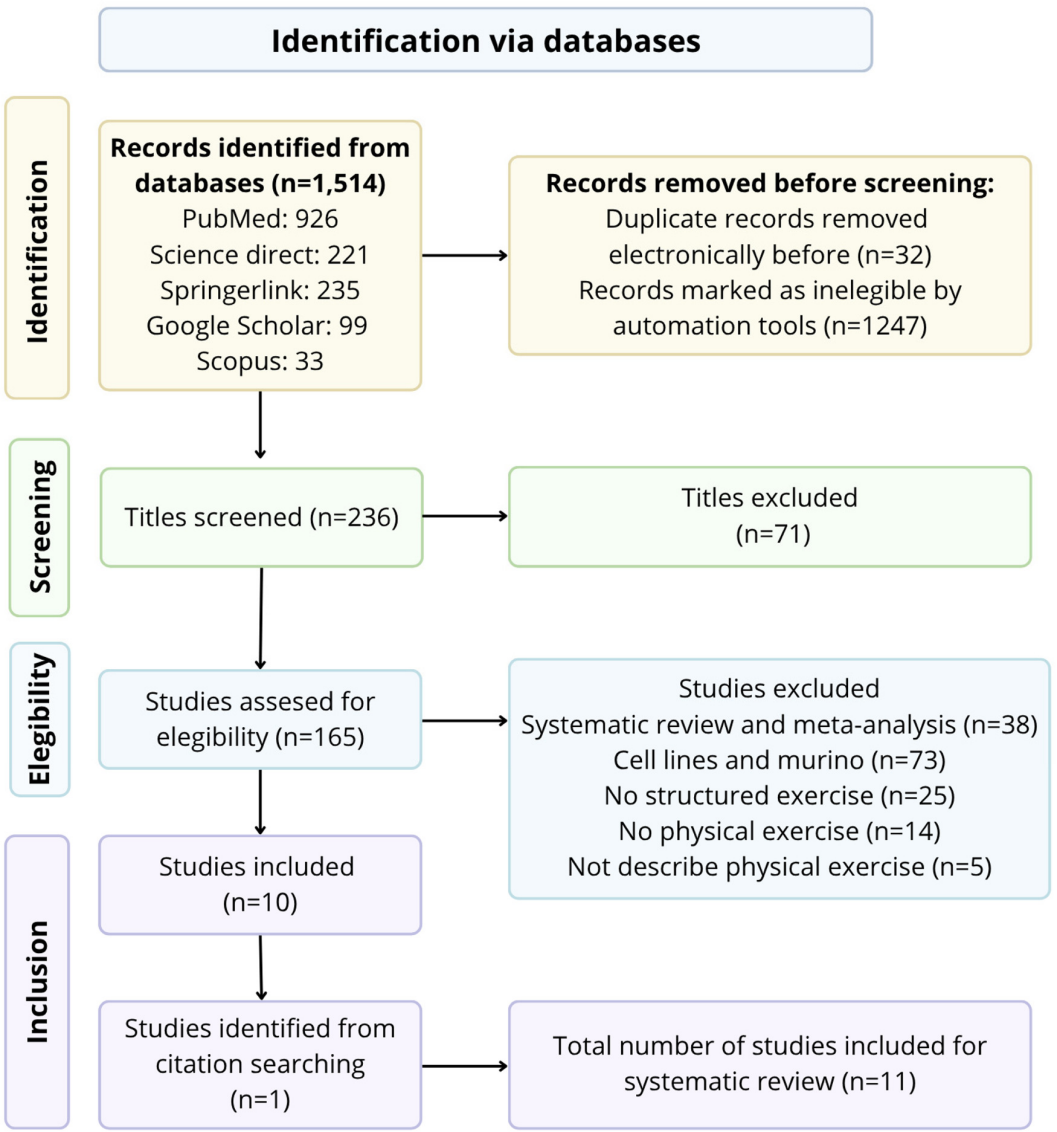
PRISMA flowchart for systematic reviews.

A total of 1,514 studies were identified by searching PubMed, Science Direct, SpringerLink, Scopus and Google Scholar databases. 32 duplicate articles were removed. After reviewing titles and abstracts, 71 articles were excluded, leaving 165 articles for full-text screening, A study was added from the references list of an article. Finally, 155 articles were excluded because they were systematic reviews or meta-analyses, cell lines or murine, did not comply as structured exercise, did not have physical activity or did not describe the details of the physical activity program. A total of 11 studies were included for this systematic review (21–31). These results are described qualitatively. The flowchart of the selection process is shown in Figure 1.

### Study characteristics

We included 11 clinical trials conducted in various countries, including Mexico, Brazil, Canada, Italy Iran, Spain, and China. Among these studies, 5 analyzed NLRP3 levels (21, 25, 26, 30, 31), 1 analyzed ASC levels (22), 1 analyzed Caspase-1 levels (31), 9 analyzed IL-1β levels (22–24, 26–31) and 2 studies analyzed IL-18 levels (24, 26). The results were derived from 593 participants (200 in the control group and 393 in the exercise group). Age ranged between 23 and 78 years old. Participants in 2 of the studies were healthy, while participants in the remaining studies reported comorbidities such as type 2 diabetes, overweight or obesity, prediabetes, hypertension, coronary artery disease and among others. Out of the 11 studies, 4 had two groups, 2 studies had three groups, 2 studies had four groups, 1 study had four groups and 2 studies had six groups. Table 1 provides a summary of the study characteristics.

**Table 1:** The effects of different types of exercise on the expression of genes involved in the NLRP3 inflammasome in adults with some metabolic conditions.

Author	Country	Type of study	Health status	Group	Age (Mean ± SD/median (25–75 percentiles)	Exercise type	Intensity	Frequency (days/week)	Duration (weeks)	Additional interventions	Evaluated parameters
Frequency of exercise ≤12 weeks											
Armannia et al. (21)	Iran	RCT	Obesity	HIIT MIT C	45.33 ± 3.55 45.17 ± 3.24 45.00 ± 2.86	AE	55%–90% HR_max_ 40–75%HR_max_	3	8	N/A	↓ NLRP3 (serum)
Gholitabar et al. (25)	Iran	RCT	Obesity	OBC RJ HV-HIIT LV-HIIT HV-HIIT + RJ LV-HIIT + RJ	48 ± 3 46 ± 4 45 ± 5 47 ± 8 49 ± 6 49 ± 4	AE + RE	80%–90%HR_max_	2	8	Royal jelly supplementation	↓ NLRP3 (serum)
Gomarasca et al. (26)	Italy	Case-control	Healthy	C IG	68 (60–78) 69 (60–78)	AE	No exercise 60%–70%HR_max_	3	12	N/A	↓ NLRP3 (serum)
Hoseini et al. (28)	Iran	RCT	T2D	AT + VIT D AT + P AT VIT D C	48.32 ± 2.23 47.13 ± 3.12 49.10 ± 1.23 48.27 ± 2.17	AE	60%–75%HR_max_	3	8	Vitamin D supplementation	↓ IL-1β (serum)
Nikseresht et al. (30)	Iran	RCT	Overweight or obesity	HC LC HC + BBR LC + BBR BBR C	48.42 ± 4.68 49.75 ± 8.13 48.75 ± 4.23 48.42 ± 4.39 52.71 ± 7.25 49.14 ± 4.29	RE	85%–95%HR_max_	2	8	Berberine supplementation	↓ NLRP3 (serum)
Frequency of exercise ≥13 weeks											
Barron-Cabrera et al. (22)	Mexico	RCT	Grade I–II obesity	CG IG	40 ± 8.1 33.6 ± 9.6	AE	No exercise 65%–75%HR_max_	3/5	16	Hypocaloric diet	↓ ASC (serum)
Zhanget al. (31)	China	RCT	Prediabetes	CG PCG PIG	59.4 ± 6.37 63.0 ± 4.72 61.1 ± 6.57	RE	60%–70%HR_max_	5	24	Yijingjing	↓ IL-1β, NLRP3 and caspase-1 (serum)

HIIT, hight-intensity interval training; MICT, moderate-intensity continuous training; C, control; OBC, obese control with placebo; RJ, royal jelly; HV-HIIT, high-volume HIIT; LV-HIIT, low-volume HIIT; HV-HIIT + RJ, high-volume HIIT with RJ; LV-HIIT + RJ, low-volume HIIT with RJ; IG, intervention group; AT + VIT D, aerobic training with vitamin D; AT + P, aerobic training with placebo; AT, aerobic training; VIT D, vitamin D; HC, high compression; LC, low compression; HC + BBR, high compression + berberine; LC + BBR, low compression + berberine; PCG, prediabetic control group; PIG, prediabetic intervention group; N/A, not applicable; AE, aerobic exercise; RE, resistance exercise; HRmax, maximum heart rate; T2D, type 2 diabetes; RCT, randomized clinical trial; ↓, down regulation.

The detailed characteristics of training for each study are presented in Tables 1, 2. The exercise intervention mainly included three types: aerobic exercise (AE), resistance exercise (RE), combined aerobic and resistance exercise (AE + RE), and isometric grip strength. Aerobic exercise included activities like walking, jogging, standard Nordic walking, cycling and, in some cases, treadmill and/or cycle ergometer use. Resistance exercise aimed to increase muscle strength through exercises such as transverse plane pushing, frontal plane pulling, squat jump, abdominal crunches, leg press, bicep curls, pectoral deck, jumping jack, sometimes using dumbbells and/or resistance bands. Isometric grip exercise involved isometric contractions using a handgrip dynamometer. The training frequency ranged from 2 to 5 sessions per week with durations ranging from 8 to 24 weeks. In all control groups, no exercise intervention was applied, and participants were instructed to continue their daily routines. In 6 out of 11 studies, participants received pharmacological, supplementation or dietary interventions to manage specific diseases.

**Table 2:** The effects of different types of exercise on cytokines involved in the NLRP3 inflammasome in adults with some metabolic conditions.

Author	Country	Type of study	Health status	Group	Age (Mean ± SD/Median (25–75 percentiles)	Exercise type	Intensity	Frequency (days/week)	Duration (weeks)	Additional interventions	Evaluated parameters
Frequency of exercise ≤12 weeks											
Cahu Rodrigues et al. (23)	Brazil	RCT	Hypertension	C IG	59 ± 2 61 ± 2	Isometric Grip	30% MVC	3	12	Antidepressive pharmacologic	=IL-1β (plasma)
Garneau et al. (24)	Canada	RCT	Coronary artery disease and T2D	HIIT-non-T2D MICT-non-T2D HIIT-T2D MICT-T2D	60.2 ± 7.8 62 ± 7.2 61.8 ± 3.6 56.8 ± 7.5	AE + RE	60%–95%HR_max_	2	12	N/A	=IL-1β and ↓ IL-18 (plasma)
Gomarasca et al. (26)	Italy	Case-control	Healthy	C IG	68 (60–78) 69 (60–78)	AE	No exercise 60%–70%HR_max_	3	12	N/A	=IL-18 and ↓ IL-1β (serum)
Hooshmand Moghadam et al. (27)	Iran	RCT	Obesity and T2D	CT S CTS C	39 ± 5	AE + RE	80%–95%HR_max_	3	12	Saffron supplementation	↓ IL-1β (serum)
Liu et al. (29)	China	RCT	Estenosis coronaria	C CAD	64.0 (61.9–66.1) 62.0 (59.5–64.5)	AE	45%–60%HR_max_	3	12	N/A	↓ IL-1β (plasma)
Nikseresht et al. (30)	Iran	RCT	Overweight or obesity	HC LC HC + BBR LC + BBR BBR CON	48.42 ± 4.68 49.75 ± 8.13 48.75 ± 4.23 48.42 ± 4.39 52.71 ± 7.25 49.14 ± 4.29	RE	85%–95%HR_max_	2	8	Berberine supplementation	↓ IL-1β (serum)
Frequency of exercise ≥13 weeks											
Barron-Cabrera et al. (22)	Mexico	RCT	Grade I–II obesity	CG IG	40 ± 8.1 33.6 ± 9.6	AE	No exercise 65%–75%HR_max_	3/5	16	Hypocaloric diet	=IL-18 (serum)
Zhang et al. (31)	China	RCT	Prediabetes	CG PCG PIG	59.4 ± 6.37 63.0 ± 4.72 61.1 ± 6.57	RE	60%–70%HR_max_	5	24	Yijingjing	↓ IL-1β (serum)

C, control; IG, intervention group; HIIT-non-T2D, hight-intensity interval training non T2D; MICT-non-T2D, moderate-intensity continuous training non T2D; HIIT-T2D, hight-intensity interval training with T2D; MICT-T2D, moderate-intensity continuous training with T2D; CT, concurrent training + placebo; S, saffron supplementation; CTS, concurrent training + saf- fron supplementation; CAD, coronary artery disease; HC, high compression; LC, low compression; HC + BBR, high compression + berberine; LC + BBR, low compression + berberine; PCG, prediabetic control group; PIG, prediabetic intervention group; N/A, not applicable; AE, aerobic exercise; RE, resistance exercise; HR_max_, maximum heart rate; T2D, type 2 diabetes; MCV, maximal voluntary contraction; RCT, randomized clinical trial; ↓, down regulation; =, not changes.

### Risk of bias of selected studies

**Figure 2:**
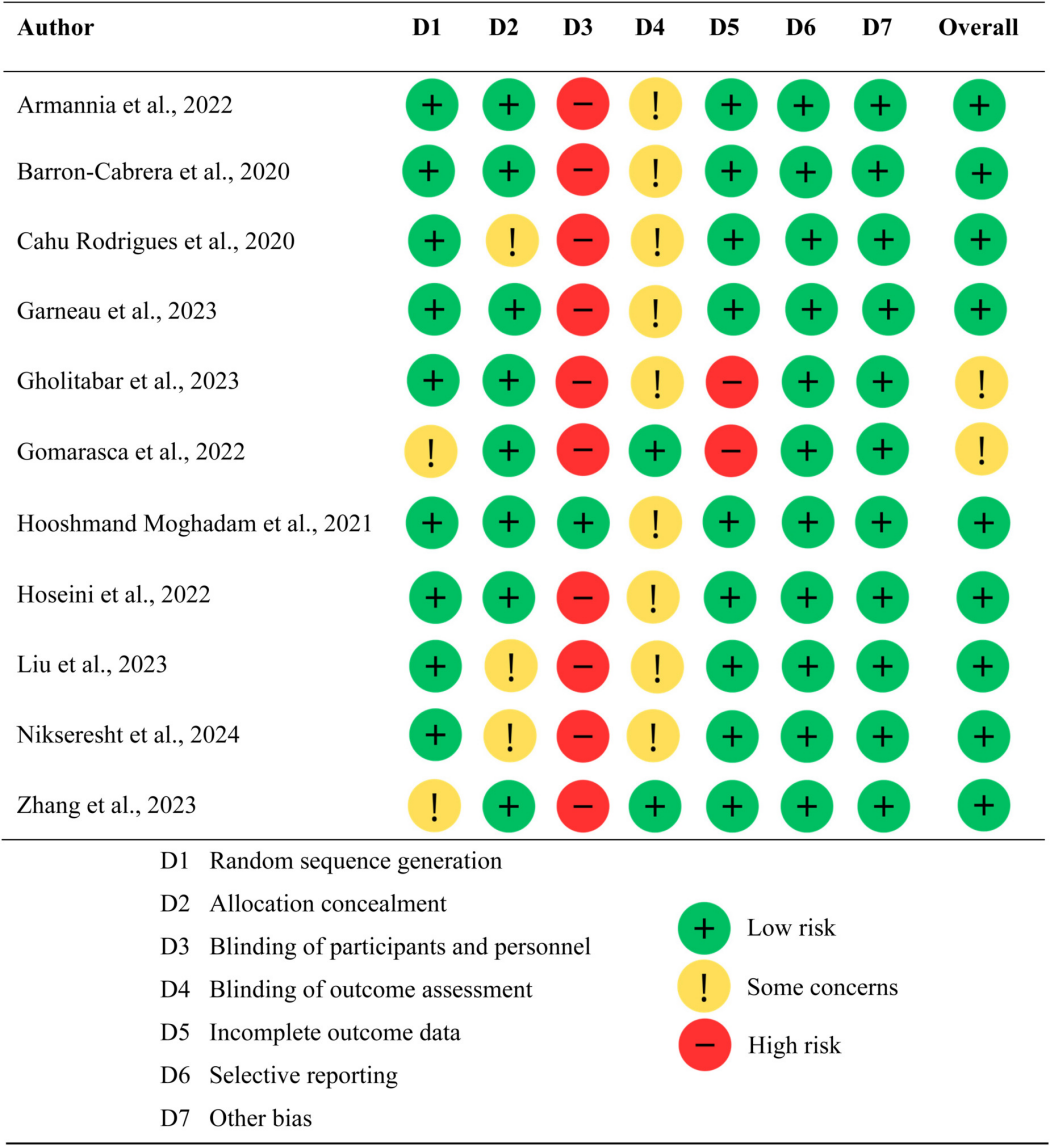
Quality assessment of studies using the cochrane risk of bias tool.

The “Cochrane Tool for Risk of Bias” (20) was employed to assess the quality of the included studies (Figure 2). Two studies did not clearly describe how the random sequence was generated. Ten studies showed a high risk of bias in participant and personnel blinding due to the nature of the exercise intervention. Nine studies did not mention details of outcome assessment blinding, while two studies had incomplete outcome data. No study had a high risk of other bias or selective reporting. According to the Cochrane tool, two studies were of medium quality (score of 3–4), and nine studies were of high quality (score of 5–7).

### Effects of physical exercise on NLRP3 inflammasome

#### Aerobic exercise

##### Frequency ≤12 weeks

Four studies assessed the effect of aerobic exercise on at least one key molecule related to the NLRP3 inflammasome. In a study conducted in elderly women, assessing the impact of moderate aerobic exercise through Nordic walking over a 12-week period on the main components of the inflammasome in blood sample. A total of 58 elderly women were randomly assigned an experimental group, which participated in a Nordic walking training program, and a control group, which did not undergo any additional activity and was instructed to maintain their usual lifestyle habits. Additionally, subgroups were created based on participants' body mass index (BMI), classifying them as normal weight and overweight/obese. The training program implemented by the experimental group consisted of three sessions per week, each lasting 60 min, comprising a 10-min warm-up, 40 min of Nordic walking, and a 10-min cool-down. Standard Nordic walking poles were used during the sessions, and the activity was supervised by a team of assistants and trainers. Nordic walking was performed at an intensity of 60%–70% of maximum heart rate. Blood samples were collected from the antecubital vein in the experimental group at different time points: baseline, before (T1-pre) and after (T2-post) the first Nordic walking session, just before the last exercise session, after 12 weeks of training (T2-pre), and immediately after the last training session (T2-post). For the control group, blood samples were collected at the beginning and end of the study. Total RNA was extracted from these blood samples and subjected to reverse transcription for subsequent real-time PCR of the NLRP3 gene, using the 2-ΔΔCq relative quantification method. Furthermore, concentrations of cytokines IL-18 and IL-1β were quantified in the blood serum. The experimental group showed a significant decrease in *NLRP3* gene expression at T2-pre compared to T1-pre. When comparing subgroups of normal weight and overweight/obesity within the experimental group, no significant differences were observed. However, the normal weight subgroup exhibited a significant increase in *NLRP3* gene expression (T1-pre vs. T1-post). Nevertheless, subsequently at 12 weeks and before starting the training session, a significant decrease in *NLRP3* expression (T1-pre vs. T2-pre) was observed in this same group. IL-18 concentrations remained unchanged in the experimental group, with no significant differences between the experimental and control groups or between normal weight and overweight/obesity subgroups. On the other hand, IL-1β cytokine experienced a decrease at the beginning of training (T1-pre vs. T1-post), increased after the first exercise period, decreased after 12 weeks of training, and increased again in the last exercise session. The main findings indicate that participation in a Nordic walking training program is associated with a reduction in inflammasome expression at rest, as well as the acquisition of a post-exercise proinflammatory response after completing the Nordic walking program. When categorizing participants based on their body mass index, it is observed that the NLRP3 gene response is more pronounced in individuals with normal weight compared to those with obesity (26).

A study conducted in adults with obesity aimed to assess the effects of two aerobic training strategies: high-intensity interval training (HIIT) and moderate-intensity continuous training (MICT). Participants were randomly assigned to one of three groups: HIIT, MICT, or a control group. The exercise groups underwent training three times per week for a period of 8 weeks. The HIIT protocol was performed at an intensity ranging from 55% to 90% of maximum heart rate (HRmax), while the MICT protocol ranged from 40% to 75% of HRmax. Each session lasted a total of 35 min and included a 5-min warm-up and a 5-min cool-down. Gene expression analysis of *NLRP3* revealed a significant downregulation in both exercise groups compared to the control group (21).

A randomized study was conducted to evaluate the combined effects of vitamin D supplementation and aerobic exercise in individuals with obesity. Participants were allocated into four groups: aerobic training plus vitamin D (AT + Vit D), aerobic training plus placebo (AT + placebo), vitamin D alone (Vit D), and control with placebo (C). The AT + Vit D and Vit D groups received a weekly dose of 50,000 IU of vitamin D, while the AT and control groups were administered a placebo identical in color, taste, and form. The aerobic exercise protocol, assigned to the AT + Vit D and AT + placebo groups, was home-based and performed three times per week over 8 weeks. Each session began with 10 min of warm-up and ended with 10 min of cool-down. The main exercise component started at 20 min per session at 60% of maximum heart rate and progressively increased to 40 min at 75% of maximum heart rate. Gene expression of *IL-1*β was assessed as the primary outcome. The findings indicated that eight weeks of aerobic exercise combined with vitamin D supplementation significantly downregulated *IL-1*β gene expression compared to the control group (28).

A study investigated the role of meteorin-like protein (Metrnl) in mitigating vascular inflammation in CAD patients post-exercise. CAD patients who underwent a 12-week moderate-intensity continuous training (MICT) regimen exhibited decreased inflammatory cytokine expression, particularly IL-1β. In fact, plasma Metrnl levels, were inversely associated with, IL-1β and CAD severity. These decreased cytokine levels were most likely due to NLRP3 inflammasome down-regulation (29).

##### Frequency ≥13 weeks

In a study of adults with obesity grade I and II were randomized into two groups. The first group underwent a nutritional program, consisting of a hypocaloric diet with a 20% reduction in total energy expenditure, calculated using the Mifflin St. Jeor formula adjusted to current weight, and a macronutrient distribution of 50% carbohydrates, 30% fats, and 20% proteins. The second group combined the hypocaloric diet with progressive moderate-intensity aerobic exercise. The main exercises focused on improving aerobic capacity through walking and jogging, functional exercise circuits, and short sprints to enhance speed. Dumbbells weighing less than 5 kg were also incorporated to promote endurance. Analysis of *ASC* gene mRNA expression from leukocytes in peripheral blood was performed using the relative quantification method 2-ΔΔcq. At baseline, no significant differences were found between the two study groups. At the final time point, a significant decrease in *ASC* gene mRNA expression was observed in the group combining diet and exercise compared to the group that only underwent dietary intervention. Additionally, a significant decrease in *ASC* gene mRNA expression was found from baseline to final time in the group that performed both diet and exercise compared to the group that only implemented the hypocaloric diet. IL-18 and IL-1β cytokines were quantified in serum; however, no significant differences were observed in their levels over time in this study (22).

Current evidence suggests that regular aerobic exercise (three times per week), performed at an intensity ranging from 40% to 90% of maximum heart rate, can exert significant anti-inflammatory effects. These effects are mediated by the downregulation of key genes associated with the NLRP3 inflammasome, particularly *ASC*, *NLRP3*, and IL-*1*β. However, these transcriptional changes are not reflected in the circulating levels of the cytokines IL-18 and IL-1β. These findings indicate that such a physical intervention constitutes an effective strategy for mitigating low-grade chronic inflammation commonly associated with metabolic diseases such as obesity and type 2 diabetes through the downregulation of the NLRP3 inflammasome. Notably, similar effects have also been observed in metabolically healthy individuals.

#### Resistance exercise

##### Frequency ≤12 weeks

Two studies evaluated the effects of resistance exercise on the NLRP3, IL-1β and caspase-1 genes, and the cytokine IL-1β. In a study conducted on elderly individuals with prediabetes, the impacts of combined Yijinjing and resistance training were investigated to determine whether a direct correlation between the NLRP3 inflammasome and symptoms associated to insulin resistance and liver damage exist. A randomized clinical trial was performed, where participants were randomly assigned to one of three following groups: a healthy control group, a prediabetes control group, and a prediabetes exercise group. The prediabetes exercise group participated in combined Yijinjing and resistance training sessions for 6 months with 5 sessions per week. The resistance part of the training involved exercises with elastic bands for 8 min, with an intensity ranging between 60% and 70% of the maximum heart rate. Each session comprised 5 min of warm-up, Yijinjing training, elastic band training, and 5 min of cooling down, with a total duration of 47–76 min per session. Concentrations of the cytokine IL-1β in serum were determined using ELISA kits. To assess NLRP3 inflammasome activity, protein expressions of the inflammasome signaling cascade, including NLRP3, IL-1β, and caspase-1, were detected in peripheral blood mononuclear cells by western blot analysis. A significant overactivation of the inflammasome was observed in the prediabetes control group compared to healthy control subjects, as indicated by the results obtained through NLRP3, caspase-1, and IL-1β protein concentrations. The serum IL-1β concentration was higher in the prediabetes control group than in control subjects. On the other hand, the gene expression of NLRP3, CASPASE-1, and IL-1β, as well as the serum IL-1β concentration, decreased significantly in the prediabetes exercise group after 6 months of combined Yijinjing and resistance training (31).

##### Frequency ≥13 weeks

In another study conducted in middle-aged overweight or obese men with prediabetes, they evaluated the effect of a low-volume HIIT training program with different levels of compression, with or without berberine supplementation, on the upregulation of NLRP3 and IL-1β non-coding RNA. Study participants were assigned to one of the following groups: high compression HIIT (HC), low compression HIIT (LC), high compression HIIT with berberine supplementation (HC + BBR), low compression HIIT with berberine supplementation (LC + BBR), exclusive berberine supplementation (BBR) and a control group (CON). The training groups performed a home HIIT protocol twice a week for 8 weeks, while the control and BBR groups maintained a sedentary lifestyle. The high-compression HIIT protocol consisted of 2–4 sets of 8 20-s sequences of physical activity, interspersed with 10 s of rest, with a 1-min rest between sets. In contrast, the low compression HIIT protocol included 20 s of rest between sequences. Participants in the HC + BBR, LC + BBR, and BBR groups received supplementation of 1,000 mg berberine in capsules daily. The results indicated that the low compression (LC) HIIT group presented a significant decrease in NLRP3 expression at the end of the intervention. In addition, all exercise groups, regardless of berberine supplementation, showed a reduction in IL-1β levels, suggesting a beneficial effect of HIIT on systemic inflammation in this population (30).

Several studies have demonstrated that progressive resistance training elicits anti-inflammatory effects by downregulating inflammasome-related gene expression, particularly NLRP3, CASPASE-1, and IL-1β. These effects are especially notable in older adults and individuals with prediabetes, overweight, or obesity. The observed molecular changes reflect a reduction in low-grade chronic inflammation, which is commonly associated with metabolic disorders and aging. A progressive resistance training program is therefore recommended as an effective strategy to modulate inflammation via the NLRP3 inflammasome pathway. Such programs should be performed 2–5 times per week for a minimum of 12 weeks, at an intensity ranging between 60% and 95% of maximum heart rate.

#### Combined aerobic and resistance exercise

##### Frequency ≤12 weeks

In a study in subjects with obesity they analyzed the effects of a high-intensity interval training (HIIT) program (TABATA) at a high and low volume, in conjunction with royal jelly supplementation. Participants were allocated into five experimental groups, a control group, and a placebo group. The training protocols were administered twice weekly over an 8-week period, while the supplementation groups received 1,000 mg of royal jelly daily for the same time. The relative expression of the NLRP3 gene was quantified using real-time polymerase chain reaction (RT-PCR). The findings indicated a significant reduction in NLRP3 expression across all intervention groups following the 8-week period, with the most pronounced decrease observed in the high-volume HIIT group receiving royal jelly supplementation (25).

Another study involving men with obesity and type 2 diabetes mellitus examined the effects of saffron supplementation combined with concurrent training (aerobic and resistance exercise). Participants were randomly assigned to one of four groups: concurrent training plus placebo (CT), saffron supplementation only (S), concurrent training combined with saffron supplementation (CTS), or a control group (CON). The training protocol consisted of four components per session: a 10-min warm-up, a resistance training (RT) circuit, an aerobic training (AT) circuit, and a 10-min cool-down. The RT component included exercises such as leg press, bench press, leg extension, lat pulldown, lying leg curl, and shoulder press. The AT protocol involved 10 1-min high-intensity intervals on a treadmill at 80%–95% of maximum heart rate (HRmax), interspersed with 1-min active recovery periods at 40%–60% of HRmax. Training intensity began at 80% HRmax in the first week and was progressively increased to 95% HRmax by the final week. Participants in the CTS group received a 100 mg capsule of pure saffron immediately after each training session and at the same time on non-training days. Those in the S group took the same daily dose of saffron at a consistent time, while participants in the CT group received an identical placebo daily. The study measured levels of the pro-inflammatory cytokine IL-1β. Results demonstrated that all three intervention groups—CT, S, and CTS—experienced a significant reduction in IL-1β concentrations compared to the control group (27).

Other study examined the effects of two types of exercise training [moderate-to-vigorous intensity continuous training (MICT) and high-intensity interval training (HIIT)] on circulating cytokines in male patients with coronary artery disease (CAD), with and without type 2 diabetes. Over 12 weeks, both MICT and HIIT led to significant reductions on IL-18, regardless of the presence of T2D or exercise modality. No significant effects were observed for IL-1β. Overall, both HIIT and MICT were effective in attenuating systemic low-grade inflammation in CAD patients, particularly those with T2D (24).

It has been demonstrated that combining aerobic and resistance exercise, as well as high-intensity interval training (HIIT), effectively reduces the expression of the NLRP3 gene and the proinflammatory cytokine IL-1β in individuals with obesity and type 2 diabetes. These anti-inflammatory effects have been observed even with a low training frequency of just two sessions per week over a period of 8–12 weeks, and were further enhanced when exercise was combined with adjunct interventions such as saffron or royal jelly supplementation. These findings suggest that implementing a combined aerobic and resistance exercise program, or a low-frequency HIIT protocol performed at least twice weekly for a minimum of 6–8 weeks, constitutes an effective strategy to reduce systemic inflammation mediated by the NLRP3 inflammasome and IL-1β, particularly in individuals with obesity and type 2 diabetes mellitus.

#### Isometric grip

##### Frequency ≤12 weeks

A randomized clinical trial aimed to determine if isometric grip reduces arterial stiffness and, in turn, reduces blood pressure. Patients with hypertension were recruited and stratified by sex and systolic blood pressure into three different groups: home isometric grip training, supervised isometric grip training, and a control group. Isometric grip training consisted of 3 sessions per week for 12 weeks. Each session involved four sets of 2-min isometric contractions with alternating hands, using a grip dynamometer at 30% of the maximum voluntary contraction, with a 60-s rest period. Participants in the control group were asked to continue with their normal dietary and physical activity habits; however, at the end of the study, they were provided with the isometric exercise program. The concentration of the cytokine IL-1β in plasma was evaluated using the Invitrogen kit. No statistically significant changes in IL-1β concentrations were found in any group (23).

The isometric training in patients with hypertension have shown that this form of exercise can confer notable cardiovascular benefits, including reductions in blood pressure and arterial stiffness. However, after 12 weeks of intervention, no significant changes were observed in plasma levels of the proinflammatory cytokine IL-1β. These findings suggest that, unlike other exercise modalities—such as aerobic, resistance, or high-intensity interval training (HIIT)—isometric handgrip training does not exert a demonstrable direct effect on NLRP3 inflammasome modulation or on systemic inflammation as measured by circulating IL-1β levels. However, further research employing this type of training is needed to enhance the robustness of the findings.

## Discussion

The findings of this systematic review indicate that physical exercise, in its various modalities, constitutes an effective intervention to downregulate inflammatory activity mediated by the NLRP3 inflammasome. This effect is primarily achieved through the reduction of gene and/or protein expression of key inflammasome components such as NLRP3, ASC, and caspase-1, as well as through decreased circulating levels of the proinflammatory cytokines IL-1β and IL-18.

**Figure 3:**
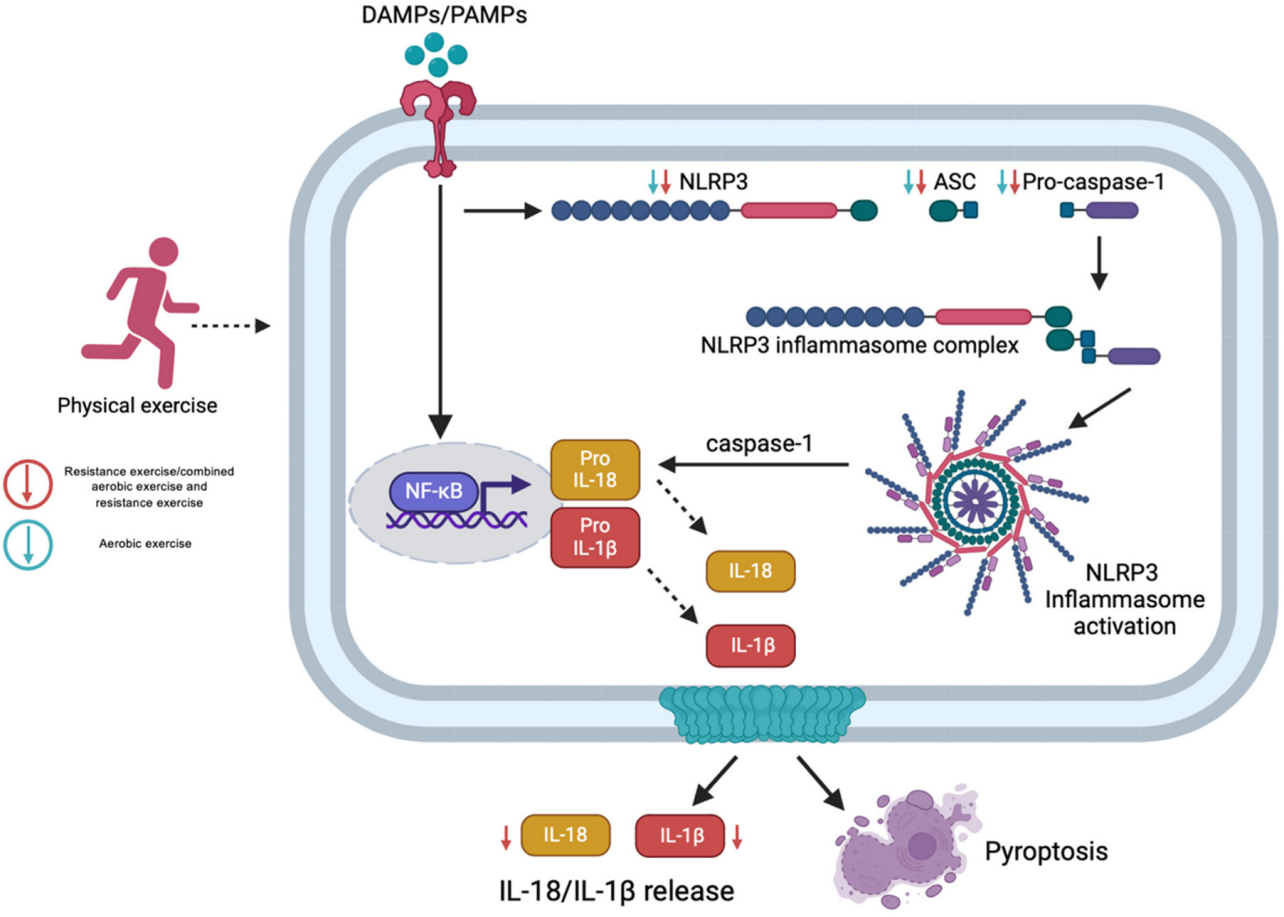
Molecular responses to NLRP3 inflammasome activation following different exercise modalities. Exercise exerts modality-specific effects on inflammation. Moderate aerobic exercise reduces NLRP3 inflammasome gene expression but shows limited impact on systemic IL-1β and IL-18. In contrast, resistance and combined training more effectively lower both gene expression and circulating cytokines, with measurable benefits within 8 weeks. Created with BioRender.com.

A key finding of this review was the modality-specific effect of exercise on inflammation. While moderate aerobic exercise consistently reduced gene expression of inflammasome-related targets, it did not consistently lower circulating IL-1β or IL-18 levels. In contrast, progressive resistance training and combined aerobic-resistance programs proved more effective in reducing both gene expression and systemic cytokine levels, with benefits evident from as early as 8 weeks of intervention (Figure 3). This discrepancy may be explained by the biphasic mechanism of inflammasome activation. The first signal (Signal 1), triggered by PAMPs and DAMPs, activates NF-κB and upregulates the transcription of NLRP3, pro-IL-1β, and pro-IL-18. However, these precursors are biologically inactive. A second signal (Signal 2) dependent on intracellular events such as oxidative stress, mitochondrial dysfunction, or ionic imbalance is required for inflammasome assembly, caspase-1 activation, and cytokine maturation. Therefore, exercise protocols that primarily affect transcriptional pathways, as seen with moderate aerobic training, may reduce gene expression without significantly affecting systemic cytokine levels, which are harder to detect due to their short half-life and localized release.

Conversely, resistance and combined training modalities induce both metabolic and mechanical stimuli, capable of simultaneously modulating Signal 1 and Signal 2, resulting in a more comprehensive suppression of inflammasome activation. This dual modulation translates into greater effectiveness in reducing both transcript and protein levels of key inflammatory mediators. Moreover, several included studies reported sustained anti-inflammatory benefits between 8 and 24 weeks of training, suggesting that structured and consistent exercise regimens can drive meaningful molecular adaptations, even in populations with elevated baseline inflammation due to metabolic conditions. In contrast, isometric handgrip exercise, while beneficial for blood pressure control, has not demonstrated significant effects on IL-1β levels, suggesting a limited capacity to modulate inflammasome-mediated inflammatory activity.

While certain limitations should be acknowledged, they do not significantly detract from the relevance and contribution of the present study. One such limitation is the heterogeneity among the included trials, which can be attributed to the relatively recent development of research exploring the effects of exercise on inflammasome activation. Consequently, the available evidence is not yet sufficient to enable a standardized comparison across different exercise modalities, metabolic conditions, and combined interventions. The inclusion of studies involving combined interventions may also enhance the effects of exercise, potentially influencing the interpretation of results and contributing to the observed variability. Additionally, some studies report only gene expression data without corresponding protein concentration measurements, which may limit the depth of interpretation. Nonetheless, despite these challenges, the study provides valuable insights and highlights important directions for future research in this emerging field.

Future research should prioritize the design and implementation of randomized controlled trials (RCTs) with robust methodological frameworks. These studies should employ standardized and supervised exercise protocols (carefully controlling for intensity, duration, and type of activity) to allow for more accurate comparisons across populations and metabolic conditions. Moreover, integrating both gene expression analyses and cytokine quantification in these trials will be essential to elucidate the upstream and downstream effects of exercise on the NLRP3 inflammasome complex and its related inflammatory mediators.

## Conclusion

Despite methodological heterogeneity among studies in terms of participant populations, intervention duration, exercise protocols, and biomarker assessments, consistent patterns emerged. Aerobic exercise, resistance exercise, and the combination of both, when performed regularly (≥8 weeks, 3 sessions per week), seems to demostrate an anti-inflammatory effect by down-regulating NLRP3, ASC, CASPASE-1, IL-1β, and IL-18, independently in individuals with disease and in healthy individuals.

Altogether, these findings underscore the role of physical exercise as a safe, non-pharmacological, and personalized strategy to counteract chronic low-grade inflammation via the NLRP3 inflammasome pathway. Resistance and combined training modalities appear particularly effective in clinical contexts where inflammation plays a central role.

## Data Availability

The original contributions presented in the study are included in the article/Supplementary Material, further inquiries can be directed to the corresponding author.
